# Effect of Constraint Loading on the Lower Limb Muscle Forces in Weightless Treadmill Exercise

**DOI:** 10.1155/2018/8487308

**Published:** 2018-04-03

**Authors:** Ning Guo, Xingyu Fan, Yuting Wu, Zhili Li, Shujuan Liu, Linjie Wang, Jie Yao, Yinghui Li

**Affiliations:** ^1^Key Laboratory for Biomechanics and Mechanobiology of Ministry of Education, School of Biological Science and Medical Engineering, Beijing Advanced Innovation Centre for Biomedical Engineering, Beihang University, Beijing 100191, China; ^2^College of Bioengineering, Chongqing University, Chongqing 400044, China; ^3^State Key Laboratory of Space Medicine Fundamentals and Application, China Astronaut Research and Training Center, Beijing 100094, China

## Abstract

Long exposure to the microgravity will lead to muscle atrophy and bone loss. Treadmill exercise could mitigate the musculoskeletal decline. But muscle atrophy remains inevitable. The constraint loading applied on astronauts could affect the muscle force and its atrophy severity. However, the quantitative correlation between constraint loading mode and muscle forces remains unclear. This study aimed to characterize the influence of constraint loading mode on the lower limb muscle forces in weightless treadmill exercise. The muscle forces in the full gait cycle were calculated with the inverse dynamic model of human musculoskeletal system. The calculated muscle forces at gravity were validated with the EMG data. Muscle forces increased at weightlessness compared with those at the earth's gravity. The increasing percentage from high to low is as follows: biceps femoris, gastrocnemius, soleus, vastus, and rectus femoris, which was in agreement with the muscle atrophy observed in astronauts. The constraint loading mode had an impact on the muscle forces in treadmill exercise and thus could be manipulated to enhance the effect of the muscle training in spaceflight. The findings could provide biomechanical basis for the optimization of treadmill constraint system and training program and improve the countermeasure efficiency in spaceflight.

## 1. Introduction

Long exposure to the microgravity will lead to the decline of musculoskeletal system, including muscle atrophy and bone loss [1]. The decrement of muscle volume and performance significantly occurs in the lower limbs [2, 3]. Previous studies have reported an 8.8% to 15.9% reduction of plantar flexor muscle volume [4], a 35%–40% reduction of neuromuscular activity, and a 17% reduction of maximal isometric torque after spaceflight [5]. The decrease of muscle force will subsequently aggravate bone loss in microgravity [6, 7]. Long duration of spaceflight will further lead to musculoskeletal injuries including bone fracture, muscle tears, and back pain [2, 8–10]. Molecular and cellular studies revealed that mechanical environment is a critical factor to maintain the musculoskeletal function [11–13]. Therefore, a properly designed loading stimulation, such as exercise, could help counteract the negative effect of microgravity.

Treadmill exercise could mitigate the musculoskeletal weakening to some extent. Currently, the used exercise devices in the International Space Station (ISS) include treadmill, bicycle ergometer, and resistance exercise device. Among these countermeasures, a significant correlation between treadmill training intensity and the muscle mass maintenance was observed, and muscle losses of astronauts with high-volume treadmill exercise were about 59% less than those with low-volume treadmill exercise [3, 14]. During the training, astronauts have to be restrained with bungee cords, which provide the vertical loading instead of the gravity effect. Increasing the constraint force may mitigate the musculoskeletal decline but can cause discomfort and local injury at the harness contact regions [15]. Constraint force of 70% to 80% body weight was usually applied according to individual experience, which may limit the efficiency of the treadmill countermeasure [3, 15]. An optimal constraint system is desired to reduce the risk of local discomfort and increase the mechanical stimulation on the musculoskeletal system. However, the quantitative relationship between treadmill constraint system and in situ force in bones and muscles remains unclear.

Numeric musculoskeletal model combined with motion capture equipment could be used to estimate the in situ load on the bone and muscle during treadmill exercise. The muscle force and joint kinetics could be calculated from kinematical data with the inverse dynamic analysis. Then, the stress of the bone under muscle forces could be calculated with the finite element simulation [16, 17]. The methodology has been applied in sport, rehabilitation, and exoskeleton design [18–21]. Quantification of musculoskeletal loading in treadmill exercise could provide biomechanical basis for the optimization of training system and improve the countermeasure efficiency.

This study aims to investigate the influence of the constraint loading mode on the lower limb muscle force in treadmill training at weightlessness. An inverse dynamic musculoskeletal model was applied to simulate the treadmill exercise. Five loading modes of the constraint system were analyzed. Due to the lack of kinematical data at weightlessness, the kinematical data of treadmill exercise at gravity was used. To minimize the result deviation caused by this simplification, the sum of the constraint loadings was assumed to be equal to the body weight.

## 2. Materials and Methods

### 2.1. Participants

Eight healthy and physically active participants volunteered to take part into the study (5 males and 3 females, age 20 ± 2, height 1.74 ± 0.16 m, and weight 63.5 ± 20 kg). Volunteers were recruited among university students. The study was approved by the local ethics board and every subject signed an informed consent before performing the trials.

### 2.2. Motion Capture Experiment

**Figure 1:**
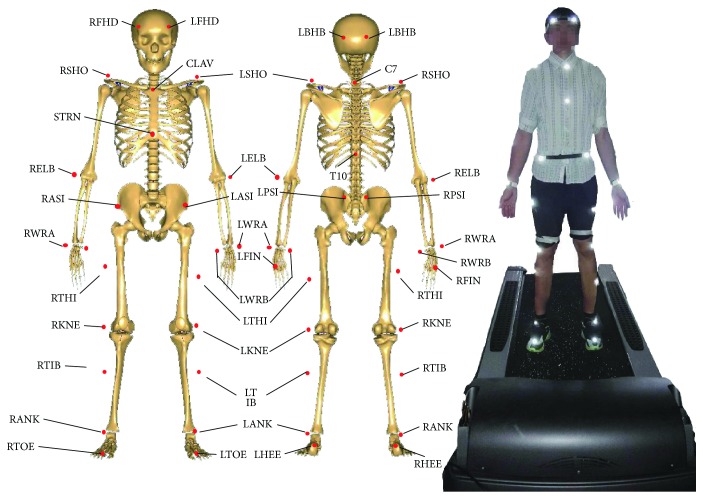
The locations of markers for motion capture experiment. Totally, 34 markers were fixed on the body during the treadmill exercise. The following is the meanings of the abbreviation for the markers' anatomic locations. R(L)FHD: right (left) front head; R(L)BHD: right (left) back head; R(L)SHO: right (left) shoulder; C7: 7th cervical; T10: 10th thoracic; CLAV: clavicle; STRN: sternum; R(L)ASI: right (left) anterior superior iliac; R(L)PSI: right (left) posterior superior iliac; R(L)ELB: right left elbow; R(L)WRA(B): right (left) wrist A(B); R(L)FIN: right (left) finger; R(L)THI: right (left) thigh; R(L)KNE: right (left) knee; R(L)TIB: right (left) tibia; R(L)ANK: right (left) ankle; R(L)TOE: right (left) toe; R(L)HEE: right (left) heel.

The subjects' kinematical information was recorded with the motion capture system, Vicon (Vicon, Oxford Metrics Ltd., UK). 34 reflective markers were attached to the bony landmarks of the subject (Figure 1). The markers' spatial coordinates were captured by 8 cameras with the sampling frequency of 100 Hz. Cameras were fixed on the wall to avoid vibration interference. Subjects were required to run on the treadmill with the speed of 1.5 m/s, which was a routine speed in the astronaut training program [10]. After the subject adapted to the treadmill speed (30 seconds), the motion information was recorded for 30 seconds. 10 stable full gait cycles were extracted from each measurement for muscle analysis. In the present study, one gait cycle was defined as the period between two adjacent left foot landings. Each subject was measured for 3 times, with one-minute interval. To eliminate the influence of the shoes on the gait, subjects were required to wear the same type of shoes with proper sizes.

### 2.3. Inverse Dynamic Model of Human Musculoskeletal System

**Figure 2:**
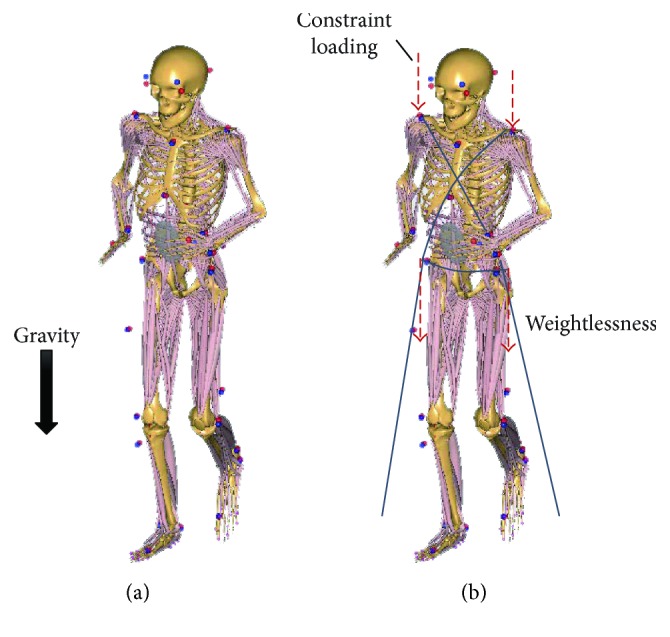
Inverse dynamic model of human musculoskeletal system and the diagram of loading conditions. The lower limbs of the model contain 6 joints and 318 muscles. (a) The model was in the condition of normal gravity. (b) The model was in the condition of weightlessness; the body was restrained with the bungee cords, which provide the vertical loading instead of the gravity effect.

A human musculoskeletal model was developed with the inverse dynamic software, AnyBody Managed Model Repository (AnyBody Technology, Denmark). The lower limbs of the model contain 6 joints and 318 muscles. The accuracy of the muscle force prediction model was validated in the previous study. Compared with the *in vivo*-measured maximal voluntary moment, the calculated data was within the 95% confidence interval [22]. The weight, height, thigh length, shank length, foot length, and pelvis width of the model were set according to the subject. The model was driven by the motion information of the markers from motion capture experiment (Figure 2(a)). The ground reaction force (GRF) during running was calculated with a GRF prediction module [23, 24].

### 2.4. Constraint Methods

In the weightless condition, bungee cords were applied instead of gravity to constrain the body on the treadmill during exercise. Previous studies have suggested that providing a constraint force equal to gravity could better prevent musculoskeletal decline in spaceflight [14, 25]. Therefore, in the present study, the resultant force of the bungee cords was assumed to be constantly equal to gravity. The constraint forces of eight loading modes were applied on the shoulder (bilateral acromion) and waist (bilateral anterior superior spine) (Figure 2(b)). The influence of the constraint loading modes on lower limb muscle forces was analyzed. Eight loading modes of constraint system were analyzed (Table 1).

In the weightless musculoskeletal model, the gravity was set to zero. The experiment on NASA KC-135 research aircraft had reported that the gait at weightlessness approached to the gait at gravity when the resultant constraint force increased to body weight on earth [26]. Therefore, the kinematical data of treadmill exercise at gravity was also used to drive the musculoskeletal model at weightlessness. A quadratic muscle recruitment method was implemented in the present study.

### 2.5. Muscle Force and Data Processing

The following muscles were analyzed in the present study: biceps femoris, gastrocnemius, vastus, soleus, and rectus femoris, which were primary active muscles in treadmill exercise. The muscle forces in the full gait cycle were calculated. To normalize the data for each trail, the muscle force was divided by the subject's body weight. Then, the normalized muscle forces of all subjects' trails were averaged. A Two-way random average measure intraclass correlation coefficient (ICC (2, k)) was used to assess the reliability of the motion capture and muscle force calculation. Values greater than 0.75 indicate desirable repeatability of the methodology. Statistical software SPSS (IMB, US) was used for the data analysis.

### 2.6. Comparison with Electromyography (EMG)

To evaluate the reliability of the muscle force calculation, the calculated muscle forces in treadmill exercise at gravity were compared with the muscle EMG data in the literature [27]. The magnitude of the EMG could not sensitively reflect the muscle force in different muscles and action types; therefore, the correlation between the timing of peak EMG and the timing of peak muscle force in the full gait cycle was calculated to evaluate the validity of the muscle force calculation with the statistical software SPSS (IMB, US). The Spearman correlation factor was calculated. A correlation coefficient different from 0 and a significant level (*p* value) < 0.05 indicates a considerable correlation.

## 3. Result

### 3.1. Relationship between Muscle Force and EMG

**Figure 3:**
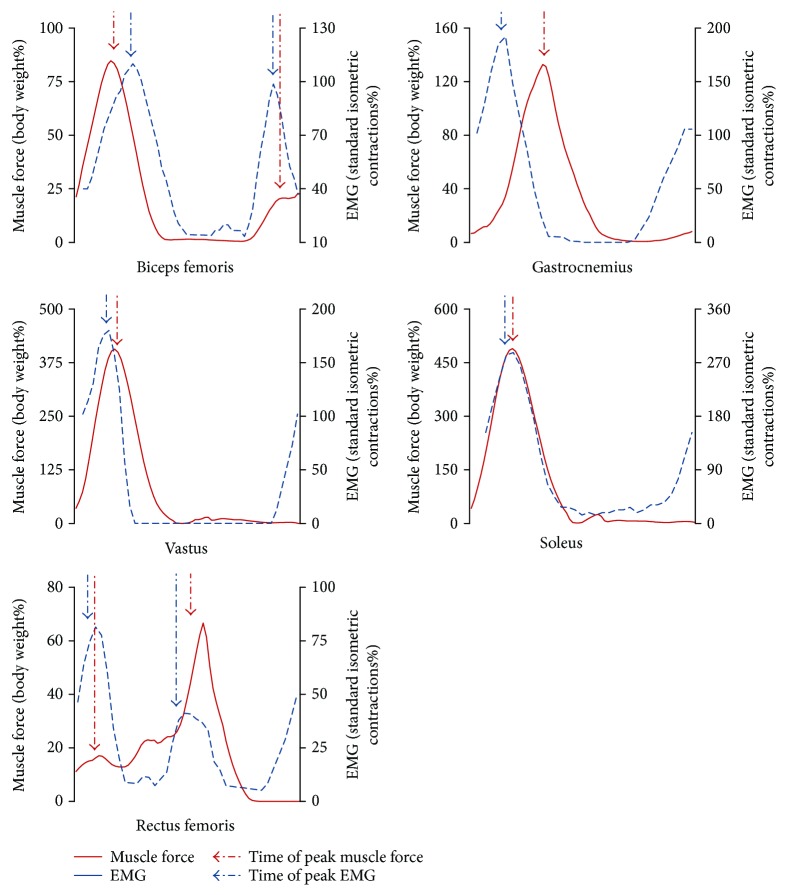
The force and EMG of the biceps femoris, gastrocnemius, rectus femoris, soleus, and vastus in a full gait cycle. The muscle forces were normalized with body weight; the EMG magnitudes were normalized with the standard isometric contractions. Since EMG magnitude could not sensitively reflect the muscle force, the correlation between the timing of peak EMG and the timing of peak muscle force was calculated to evaluate the validity of the muscle force calculation.

The normalized force and EMG of the biceps femoris, gastrocnemius, vastus, soleus, and rectus femoris in the full gait cycle were shown in Figure 3. The timing of the peak muscle force was positively correlated with the timing of the peak EMG (*r* = 0.757^∗^, *p* = 0.049), which provided the validity of the muscle force calculation (Table 2). Furthermore, the ICC (2, k) results of the muscle forces in gravity and each loading mode were greater than 0.75, which indicate desirable repeatability of the methodology (Supplementary Material, Tables S1 and S2).

### 3.2. Influence of Loading Mode on Muscle Force

Although the resultant constraint loading was constantly equal to the body weight at gravity, the maximum forces of biceps femoris, gastrocnemius, and vastus at weightlessness (all five modes) were greater than those at gravity (paired-samples *t*-test, *p* < 0.01). Furthermore, the muscle forces changed with the constraint modes. The average and the deviation of all normalized muscle forces were contained in Supplementary Material (Figures S1–S5, Tables S3–S7).

**Figure 4:**
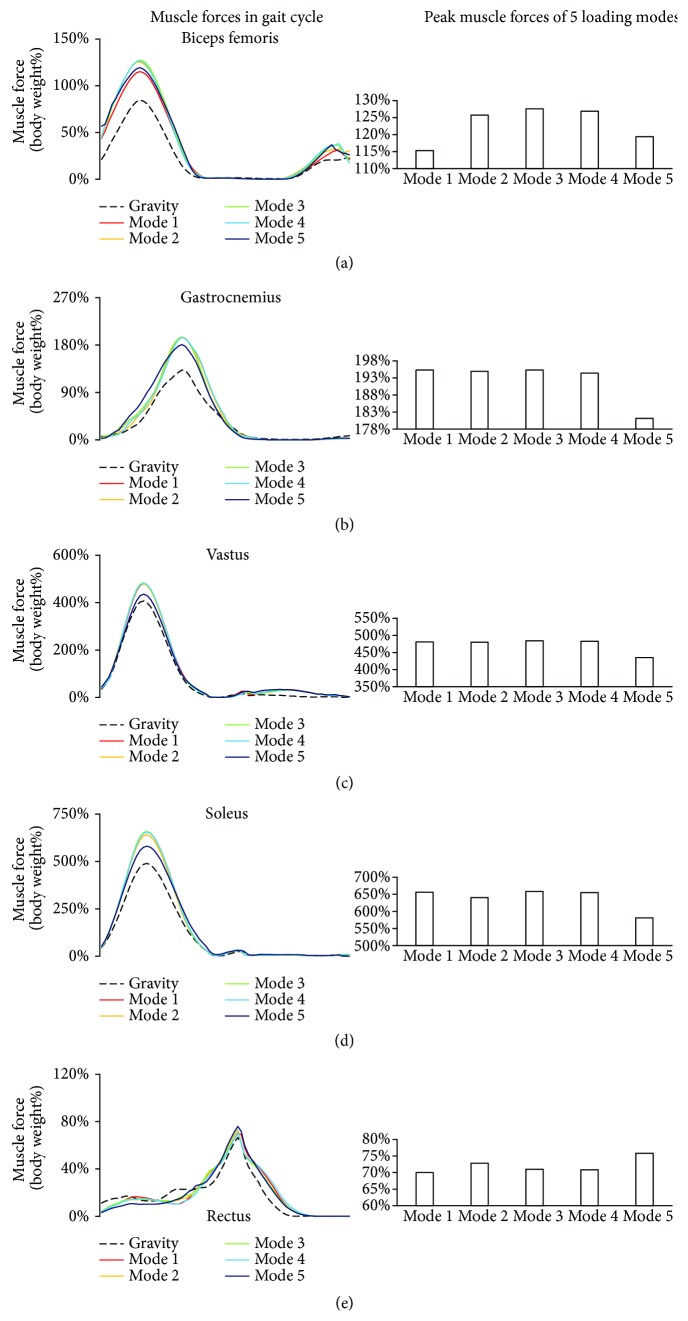
Muscle forces at gravity and five loading modes at weightlessness. The muscle forces were normalized with the body weight. For each muscle, the curve of the forces in the full gait cycle under 6 loading conditions was compared. Muscle forces at weightless condition were greater than those at gravity. (a) Biceps femoris; (b) gastrocnemius; (c) vastus; (d) soleus; (e) rectus femoris.

The biceps femoris was activated in both stance and swing phases; a larger peak muscle force occurred in the stance phase, and a smaller peak muscle force occurred in the swing phase (Figure 4(a)). With the constraint loading migrating from shoulder to waist, the maximum muscle force firstly increased and then decreased, ranging from 115% to 128% of body weight.

Gastrocnemius was activated at the end of the stance phase and the beginning of the swing phase, which was a result of flexion of the knee when lifting the leg. Only one peak was observed in the gastrocnemius force curve (Figure 4(b)). With the constraint loading migrating from shoulder to waist, the peak muscle forces of loading modes 1 to 4 were similar, while minimum peak muscle force occurred at loading mode 5 (shoulder load was 0% of body weight; waist load was 100% of body weight).

**Figure 5:**
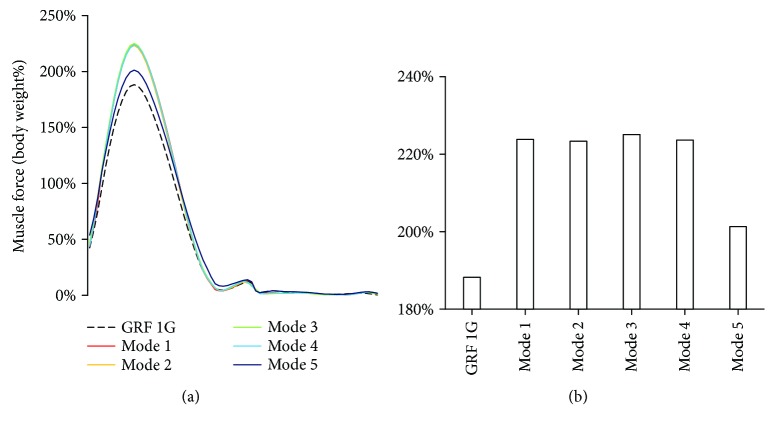
GRFs at gravity and five loading modes at weightlessness. The GRF was normalized with the body weight. The maximum GRFs at weightless condition were greater than those at gravity. (a) GRF curves in the full gait cycle under different loading conditions. (b) The maximum GRFs under different loading conditions.

The trends of vastus and soleus forces in the gait cycle were similar. The vastus force was lower than soleus. The peak muscle forces occurred in the stance phase (Figures 5(c) and 5(d)). With the constraint loading migrating from shoulder to waist, the peak muscle forces of loading modes 1 to 4 were similar, while minimum peak muscle force occurred at loading mode 5.

In the rectus femoris, the peak muscle force occurred in the swing phase (Figure 4(e)). With the constraint loading migrating from shoulder to waist, the maximum muscle force changed slightly in loading modes 1 to 4 and decreased in loading mode 5.

### 3.3. Influence of Loading Mode on GRF

The GRF occurred only in stance phase because there is no contact with the ground during the swing phase. Only one peak of GRF was observed in the stance phase, inferring the non-heel-strike running in the present study (Figure 5(a). The peak GRF at gravity was 188% of body weight, which was lower than that at weightlessness (paired-samples *t*-test, *p* < 0.01). Among the loading modes at weightlessness, the minimum peak GRF occurred at loading mode 5. Peak GRFs at loading modes 1 to 4 were similar (Figure 5(b), Supplementary Material, Table S8).

## 4. Discussion

In the present study, a significant correlation (*r* = 0.757^∗^, *p* = 0.049) between the timings of peak muscle force and EMG was observed in the full gait cycle. These results provided the validity of the muscle force calculation. Previous studies on the treadmill constraint system mainly focused on the relationship between the resultant constraint loading and GRF, and the GRF was often regarded as an index of exercise strength. However, the present results indicated that although the resultant constraint loading was constantly equal to body weight, the GRF changed with the constraint loading modes. Minimum GRF occurred at loading mode 5 (shoulder load was 0% of body weight; waist load was 100% of body weight). Furthermore, although the GRF changed slightly in constraint loading modes 1 to 4, the muscle forces also changed with the constraint loading modes. These findings implied that resultant constraint force and GRF could not precisely reflect the muscle activity strength. The positions of the constraint loadings will influence the muscle forces in treadmill exercise, thus should be carefully considered in the optimization of exercise and the device design.

The forces of vastus and soleus had the similar trends as the GRF in the stance phase of the gait cycle, since they were all activated in this phase (Figure 4). The biceps femoris had the peaks in both the stance phase and swing phase, which correspond to its function; extension of hip joint in the stance phase; and flexion of knee joint in the swing phase. Half of the gastrocnemius muscle force was in the stance phase, and the other half was in the swing phase, which were a result of flexion of the knee when lifting the leg. Rectus femoris force was quite different among the subjects. This phenomenon may be related to the personalized exercise habits and needs to be further studied.

**Figure 6:**
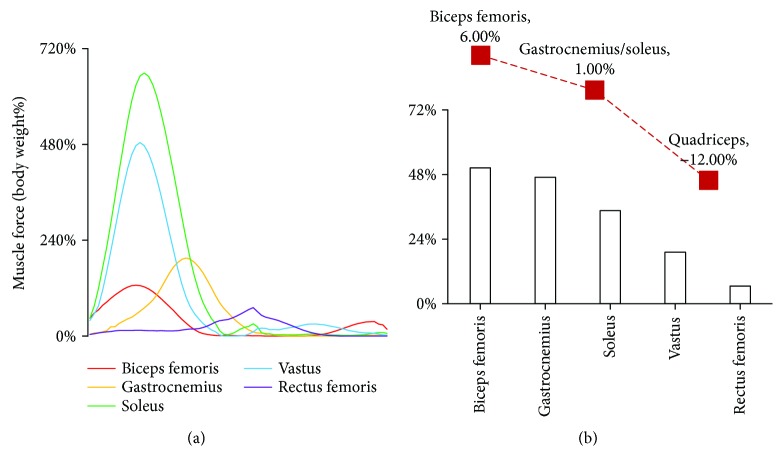
Muscle forces at weightlessness compared with those at gravity. (a) The curves of the five muscle forces at constraint loading mode 3; (b) the percent change in muscle forces based on the forces at gravity. The red curve is the postflight muscle concentric strengths compared with the preflight from NASA report [28]. The results were in agreement with the muscle atrophy observed in the astronaut.

Under the constraint loadings at weightlessness, the maximum forces of the biceps femoris, gastrocnemius, and vastus at weightlessness (all five modes) were significantly greater than those at gravity (*p* < 0.01). Take constraint loading mode 3, for example, the peak muscle forces from high to low is as follows: soleus, vastus, gastrocnemius, biceps femoris, and rectus femoris (Figure 6(a)). However, based on the muscle force at gravity, the percent change in muscle forces from high to low is as follows: biceps femoris, gastrocnemius, soleus, vastus, and rectus femoris. The results were consistent with the muscle strength change after spaceflight (Figure 6(b)) [28]. In the biceps femoris, gastrocnemius, soleus, and vastus, maximum muscle force occurred at constraint loading mode 3 (loadings applied on the shoulder and waist were 50% of the body weight). While in the rectus femoris, maximum muscle force occurred at constraint loading mode 5 (waist load was 100% of the body weight). The findings indicated that the constraint loading mode could be manipulated to enhance the effect of muscle training at spaceflight.

The calculated curve of GRF in the gait cycle was consistent with the experimental data in the literature [29], which also provided a validation for the model calculation. Previous study has reported that the running speed had an impact on the GRF. The curve of GRF had two peaks in the stance phase when running at low speed, corresponding to heel strike and toe off. With increasing the speed, the GRF peak of heel strike will disappear [30]. A relatively high speed was applied in the treadmill countermeasure, which may lead to non-heel-striking running. Therefore, both the running speed and constraint loading mode can affect the muscle forces during treadmill exercise. The loading configuration (loading magnitude, direction, loading positions, etc.) should be adaptive to the running speed in the future design of constraint system.

The present study has some limitations. First, the resultant constraint loading was constantly equal to the body weight. The current constraint bungee cord in space flight is an elastic material, implying that the constraint loading changes linearly with the body height during running. However, previous studies proposed that a constant constraint loading may better prevent the musculoskeletal decline in spaceflight [14, 25], which could be achieved by manipulating the loading devices. Second, the kinematical data of treadmill exercise at gravity was also used to drive the musculoskeletal model at weightlessness, because weightless experiment has reported that the gait at weightlessness approached to the gait at gravity when the resultant constraint force increased to body weight on earth. And the influence of the constraint loading on the body kinematics would be investigated with the supine treadmill experiment in our future study.

## 5. Conclusion

The study indicated that maximum forces of the biceps femoris, gastrocnemius, and vastus under five loading modes at weightlessness were significantly greater than those at gravity (*p* < 0.01). The percentage changes in different muscle forces were in agreement with the muscle atrophy observed in astronauts. The results further revealed that the resultant constraint force and GRF could not precisely reflect the muscle activity strength. The constraint loading mode had an impact on the muscle forces in treadmill exercise, thus could be manipulated to enhance the effect of the muscle training at spaceflight.

## Figures and Tables

**Table 1:** The constraint loading applied on the shoulder and waist.

	Mode 1	Mode 2	Mode 3	Mode 4	Mode 5
Shoulder loading	1 BW	2/3 BW	1/2 BW	1/3 BW	0 BW
Waist loading	0 BW	1/3 BW	1/2 BW	2/3 BW	1 BW

BW: body weight.

**Table 2:** The timings of the peak muscle forces and the peak EMGs in the full gait cycle.

	Biceps femoris (1st peak)	Biceps femoris (2nd peak)	Gastrocnemius	Rectus femoris (1st peak)	Rectus femoris (2nd peak)	Soleus	Vastus
Force	15%	90%	32%	10%	55%	18%	17%
EMG	17%	86%	12%	7%	49%	10%	7%

## Supplementary Materials

Supplementary Materials Figure S1: average and deviation of biceps femoris force in gait cycle in gravity and 5 loading modes. Figure S2: average and deviation of gastrocnemius force in gait cycle in gravity and 5 loading modes. Figure S3: average and deviation of vastus force in gait cycle in gravity and 5 loading modes. Figure S4: average and deviation of soleus force in gait cycle in gravity and 5 loading modes. Figure S5: average and deviation of rectus force in gait cycle in gravity and 5 loading modes. Table S1: ICC analysis in gait cycles. The table shows ICC analysis of each subject and five muscle force and GRF in gravity and 5 modes. Table S2: ICC analysis in 8 subjects. The table shows ICC analysis of 5 modes and gravity and 5 muscle force and GRF. Table S3: paired-samples *t*-test matrix of max biceps femoris force between different loading conditions. Table S4: paired-samples *t*-test matrix of max gastrocnemius force between different loading conditions. Table S5: paired-samples *t*-test matrix of max vastus force between different loading conditions. Table S6: paired-samples *t*-test matrix of max soleus force between different loading conditions. Table S7: paired-samples *t*-test matrix of max rectus femoris force between different loading conditions. Table S8: paired-samples *t*-test matrix of max GRF between different loading conditions.
