# Supplementary material for: Effect of Constraint Loading on the Lower Limb Muscle Forces in Weightless Treadmill Exercise
Source: J Healthc Eng. 2018 Apr 3;2018:8487308. doi: 10.1155/2018/8487308 (PMC5903329; doi:10.1155/2018/8487308)
Supplement: Supplementary Materials — Figure S1: average and deviation of biceps femoris force in gait cycle in gravity and 5 loading modes. Figure S2: average and deviation of gastrocnemius force in gait cycle in gravity and 5 loading modes. Figure S3: average and deviation of vastus force in gait cycle in gravity and 5 loading modes. Figure S4: average and deviation of soleus force in gait cycle in gravity and 5 loading modes. Figure S5: average and deviation of rectus force in gait cycle in gravity and 5 loading modes. Table S1: ICC analysis in gait cycles. The table shows ICC analysis of each subject and five muscle force and GRF in gravity and 5 modes. Table S2: ICC analysis in 8 subjects. The table shows ICC analysis of 5 modes and gravity and 5 muscle force and GRF. Table S3: paired-samples t-test matrix of max biceps femoris force between different loading conditions. Table S4: paired-samples t-test matrix of max gastrocnemius force between different loading conditions. Table S5: paired-samples t-test matrix of max vastus force between different loading conditions. Table S6: paired-samples t-test matrix of max soleus force between different loading conditions. Table S7: paired-samples t-test matrix of max rectus femoris force between different loading conditions. Table S8: paired-samples t-test matrix of max GRF between different loading conditions. [file 8487308.f1.pdf]

Supplementary Material

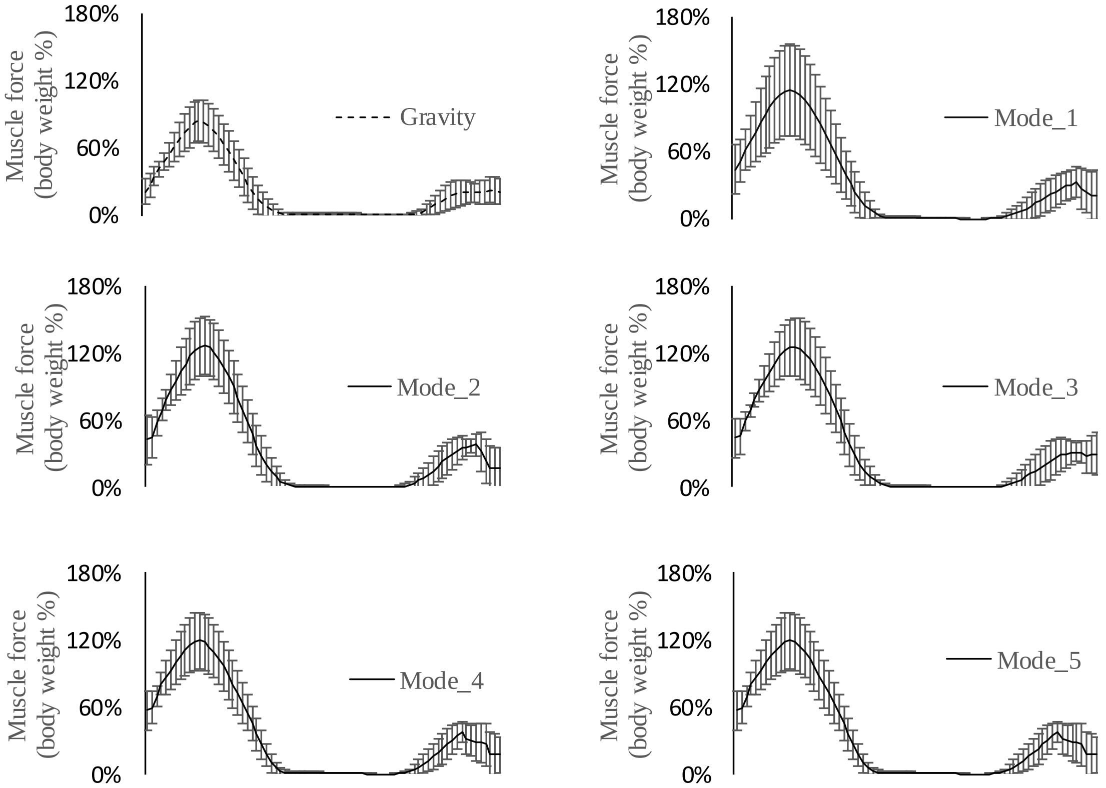

Fig. S1. Average and deviation of Biceps femoris force in gait cycle in gravity and 5 loading modes

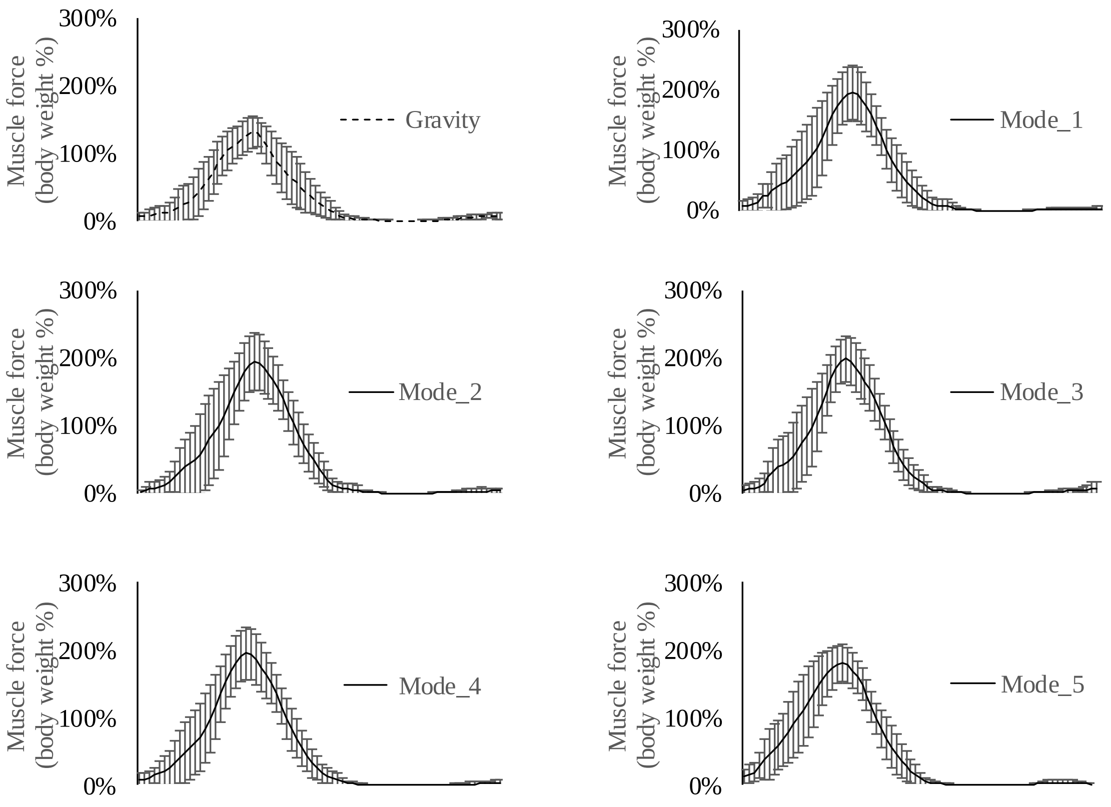

Fig. S2. Average and deviation of Gastrocnemius force in gait cycle in gravity and 5 loading modes

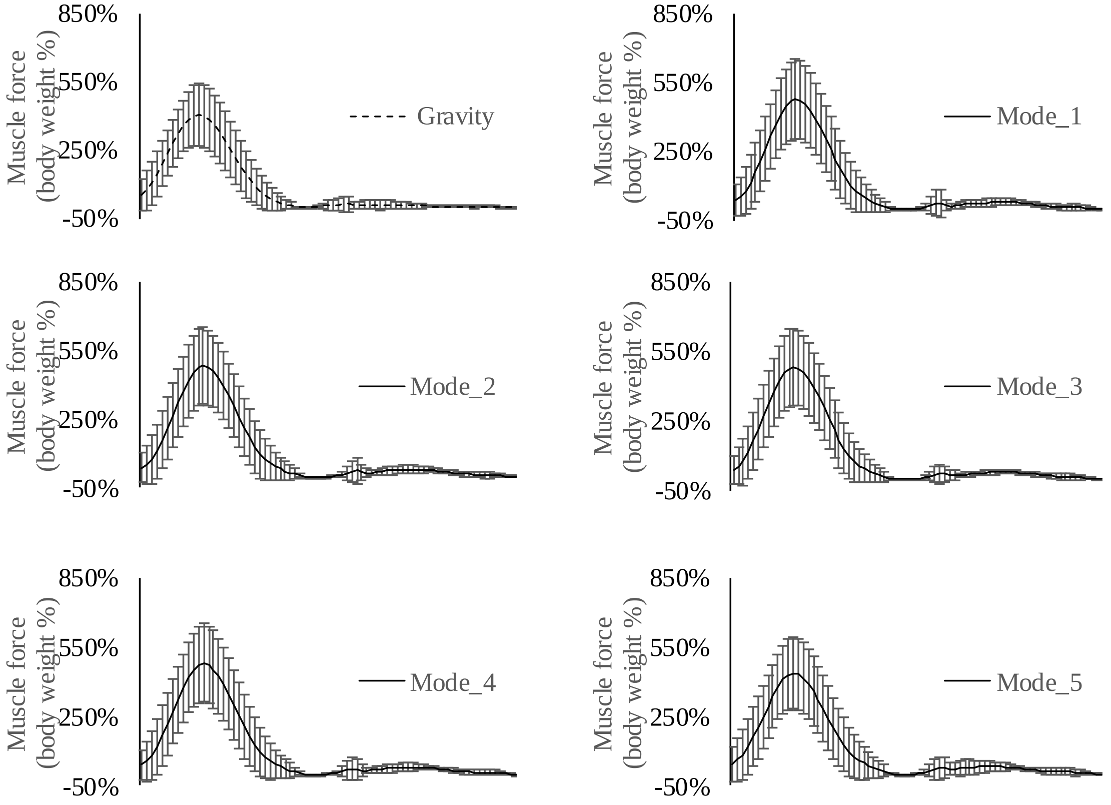

Fig. S3. Average and deviation of Vastus force in gait cycle in gravity and 5 loading modes

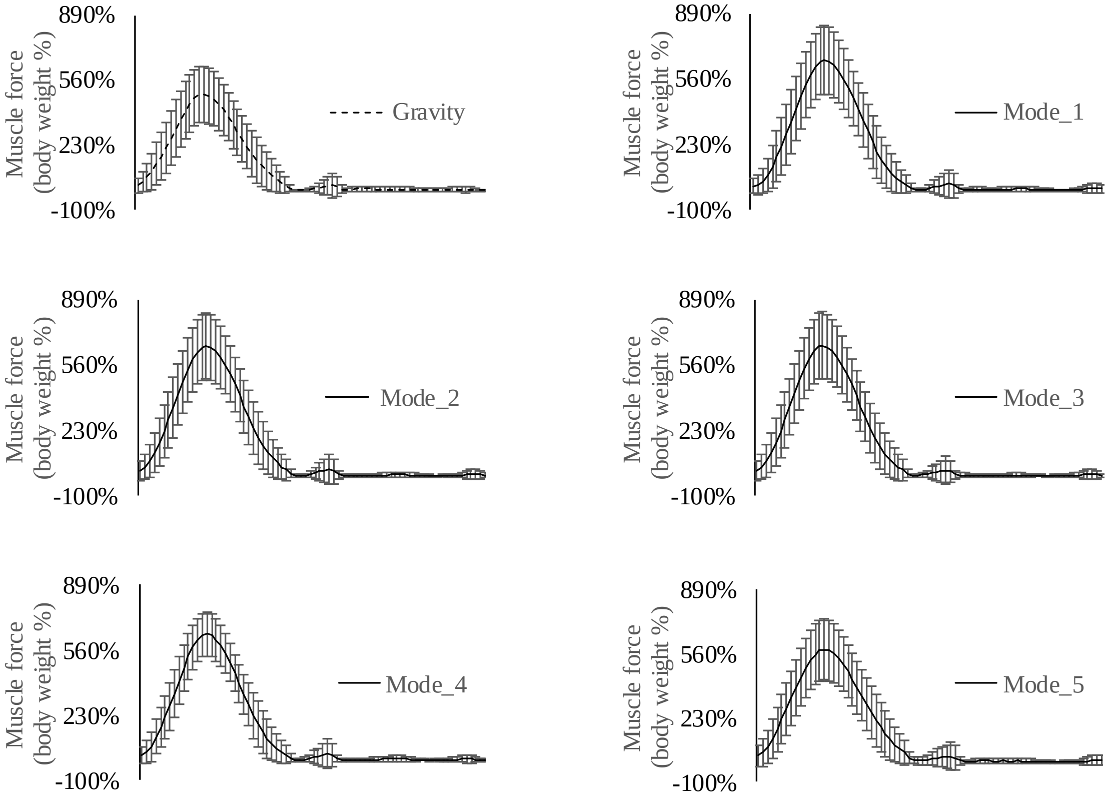

Fig. S4. Average and deviation of Soleus force in gait cycle in gravity and 5 loading modes

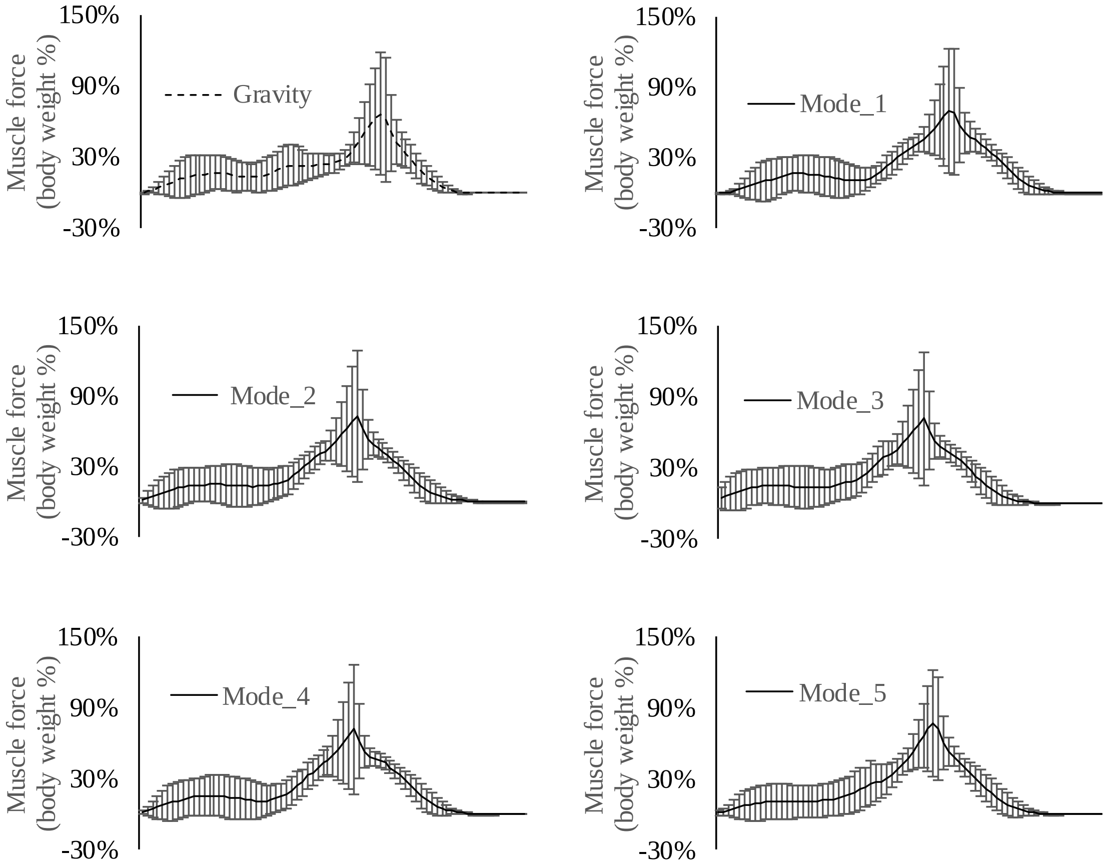

Fig. S5. Average and deviation of Rectus force in gait cycle in gravity and 5 loading modes

Table S1. ICC analysis in gait cycles

	Subject1	Subject2	Subject3	Subject4	Subject5	Subject6	Subject7	Subject8
**Biceps femoris -Gravity**	0.998	0.985	0.995	0.994	0.993	0.997	0.997	0.994
**Biceps femoris-Mode1**	0.992	0.986	0.996	0.997	0.996	0.998	0.998	0.994
**Biceps femoris-Mode2**	0.979	0.987	0.996	0.995	0.996	0.998	0.998	0.994
**Biceps femoris-Mode3**	0.996	0.987	0.996	0.996	0.996	0.998	0.997	0.994
**Biceps femoris-Mode4**	0.993	0.988	0.996	0.996	0.99	0.998	0.999	0.994
**Biceps femoris-Mode5**	0.998	0.988	0.997	0.995	0.996	0.998	0.998	0.994
**Gastrocnemius-Gravity**	0.992	0.972	0.996	0.991	0.987	0.989	0.997	0.984
**Gastrocnemius-Mode1**	0.995	0.986	0.997	0.995	0.994	0.994	0.997	0.992
**Gastrocnemius-Mode2**	0.988	0.986	0.997	0.995	0.995	0.992	0.998	0.992
**Gastrocnemius-Mode3**	0.996	0.993	0.997	0.995	0.995	0.992	0.996	0.992

**Gastrocnemius-Mode4**	0.988	0.984	0.997	0.995	0.994	0.992	0.998	0.992
**Gastrocnemius-Mode5**	0.994	0.984	0.998	0.994	0.992	0.993	0.998	0.992
**Rectus femoris-Gravity**	0.993	0.993	0.977	0.994	0.949	0.995	0.99	0.986
**Rectus femoris-Mode1**	0.978	0.991	0.98	0.99	0.989	0.995	0.992	0.984
**Rectus femoris-Mode2**	0.979	0.993	0.981	0.995	0.988	0.987	0.995	0.984
**Rectus femoris-Mode3**	0.988	0.993	0.984	0.994	0.987	0.991	0.995	0.989
**Rectus femoris-Mode4**	0.989	0.993	0.981	0.992	0.977	0.995	0.996	0.982
**Rectus femoris-Mode5**	0.994	0.992	0.982	0.995	0.97	0.996	0.996	0.982
**Soleus-Gravity**	0.998	0.99	0.998	0.999	0.998	0.998	0.995	0.995
**Soleus-Mode1**	0.992	0.996	0.998	0.998	0.999	0.998	0.996	0.995
**Soleus-Mode2**	0.992	0.996	0.998	0.999	0.999	0.999	0.996	0.995
**Soleus-Mode3**	0.995	0.995	0.998	0.999	0.999	0.999	0.996	0.995
**Soleus-Mode4**	0.993	0.996	0.998	0.999	0.995	0.999	0.996	0.995
**Soleus-Mode5**	0.998	0.994	0.998	0.999	0.999	0.998	0.996	0.994
**Vastus-Gravity**	0.999	0.983	0.996	0.999	0.992	0.999	0.996	0.996
**Vastus-Mode1**	0.991	0.982	0.996	0.999	0.993	0.999	0.996	0.995
**Vastus-Mode2**	0.991	0.991	0.996	0.999	0.993	0.998	0.996	0.996
**Vastus-Mode3**	0.994	0.982	0.997	0.999	0.993	0.999	0.996	0.996
**Vastus-Mode4**	0.991	0.991	0.996	0.999	0.984	0.998	0.996	0.996
**Vastus-Mode5**	0.999	0.982	0.994	0.999	0.99	0.998	0.996	0.996
**GRF-Gravity**	1	0.997	0.999	0.999	0.998	0.998	1	0.999
**GRF-Mode1**	0.999	0.997	0.999	0.998	0.998	0.998	1	0.999
**GRF-Mode2**	0.999	0.997	0.999	0.999	0.998	0.998	1	0.999
**GRF-Mode3**	0.999	0.996	0.999	0.999	0.998	0.998	0.999	0.999
**GRF-Mode4**	0.999	0.997	0.999	0.999	0.998	0.998	1	0.999
**GRF-Mode5**	1	0.997	0.998	0.999	0.997	0.997	1	0.999

The table shows ICC analysis of each subject and five muscle force and GRF in gravity and 5 modes.

Table S2. ICC analysis in 8 subjects

	Mode 1	Mode 2	Mode 3	Mode 4	Mode 5	Gravity
**Biceps femoris**	0.957	0.969	0.961	0.962	0.971	0.981
**Gastrocnemius**	0.976	0.978	0.982	0.984	0.983	0.973
**Rectus femoris**	0.923	0.919	0.912	0.921	0.941	0.885
**Soleus**	0.983	0.984	0.979	0.978	0.98	0.977
**Vastus**	0.969	0.972	0.97	0.97	0.966	0.974
**GRF**	0.995	0.995	0.993	0.995	0.994	0.994

The table shows ICC analysis of 5 modes and gravity and 5 muscle force and GRF.

Table S3. Paired-samples T test matrix of max biceps femoris force between different loading conditions

	Mode1	Mode2	Mode3	Mode4	Mode5	Gravity
**Mode1**	\	p=0.333	p=0.349	p=0.366	p=0.763	**p=0.038***
**Mode2**	p=0.333	\	p=0.824	p=0.816	**p=0.041***	**p=0.000****
**Mode3**	p=0.349	p=0.824	\	p=0.918	p=0.111	**p=0.000****
**Mode4**	p=0.366	p=0.816	p=0.918	\	**p=0.008****	**p=0.000****
**Mode5**	p=0.763	**p=0.041***	p=0.111	**p=0.008****	\	**p=0.000****
**Gravity**	**p=0.038***	**p=0.000****	**p=0.000****	**p=0.000****	**p=0.000****	\

* p<0.05, **p<0.01.

Table S4. Paired-samples T test matrix of max gastrocnemius force between different loading conditions

	**Mode1**	**Mode2**	**Mode3**	**Mode4**	**Mode5**	**Gravity**
**Mode1**	\	p=0.972	p=0.757	p=0.946	p=0.224	**p=0.003****
**Mode2**	p=0.972	\	p=0.854	p=0.982	p=0.536	**p=0.011***
**Mode3**	p=0.757	p=0.854	\	p=0.635	p=0.74	**p=0.001****
**Mode4**	p=0.946	p=0.982	p=0.635	\	p=0.067	**p=0.000****
**Mode5**	p=0.224	p=0.536	p=0.74	p=0.067	\	**p=0.000****
**Gravity**	**p=0.003****	**p=0.011***	**p=0.001****	**p=0.000****	**p=0.000****	\

* p<0.05, **p<0.01.

Table S5. Paired-samples T test matrix of max vastus force between different loading conditions

	**Mode1**	**Mode2**	**Mode3**	**Mode4**	**Mode5**	**Gravity**
**Mode1**	\	p=0.707	p=0.680	p=0.938	**p=0.007****	**p=0.002****
**Mode2**	p=0.707	\	p=0.809	p=0.294	**p=0.001****	**p=0.001****
**Mode3**	p=0.680	p=0.809	\	p=0.600	**p=0.003****	**p=0.001****
**Mode4**	p=0.938	p=0.294	p=0.600	\	**p=0.000****	**p=0.001****
**Mode5**	**p=0.007****	**p=0.001****	**p=0.003****	**p=0.000****	\	**p=0.022***
**Gravity**	**p=0.002****	**p=0.001****	**p=0.001****	**p=0.001****	**p=0.022***	\

* p<0.05, **p<0.01.

Table S6. Paired-samples T test matrix of max soleus force between different loading conditions

	Mode1	Mode2	Mode3	Mode4	Mode5	Gravity
**Mode1**	\	p=0.800	p=0.889	p=0.844	**p=0.001****	**p=0.000****
**Mode2**	p=0.800	\	p=0.811	p=0.849	**p=0.000****	**p=0.000****
**Mode3**	p=0.889	p=0.811	\	p=0.807	**p=0.001****	**p=0.000****
**Mode4**	p=0.844	p=0.849	p=0.807	\	p=0.403	p=0.054
**Mode5**	**p=0.001****	**p=0.000****	**p=0.001****	p=0.403	\	**p=0.000****
**Gravity**	**p=0.000****	**p=0.000****	**p=0.000****	p=0.054	**p=0.000****	\

* p<0.05, **p<0.01.

Table S7. Paired-samples T test matrix of max rectus femoris force between different loading conditions

	Mode1	Mode2	Mode3	Mode4	Mode5	Gravity
**Mode1**	\	p=0.283	p=0.919	p=0.805	p=0.484	p=0.271
**Mode2**	p=0.283	\	p=0.105	p=0.168	p=0.685	p=0.207
**Mode3**	p=0.919	p=0.105	\	p=0.878	p=0.513	p=0.287
**Mode4**	p=0.805	p=0.168	p=0.878	\	p=0.514	p=0.327
**Mode5**	p=0.484	p=0.685	p=0.513	p=0.514	\	**p=0.004****
**Gravity**	p=0.271	p=0.207	p=0.287	p=0.327	**p=0.004****	\

* p<0.05, **p<0.01.

Table S8. Paired-samples T test matrix of max GRF between different loading conditions

	Mode1	Mode2	Mode3	Mode4	Mode5	Gravity
**Mode1**	\	p=0.838	p=0.797	p=0.740	**p=0.000****	**p=0.000****
**Mode2**	p=0.838	\	p=0.750	p=0.684	**p=0.000****	**p=0.000****
**Mode3**	p=0.797	p=0.750	\	p=0.677	**p=0.001****	**p=0.000****
**Mode4**	p=0.740	p=0.684	p=0.677	\	**p=0.000****	**p=0.000****
**Mode5**	**p=0.000****	**p=0.000****	**p=0.001****	**p=0.000****	\	**p=0.000****
**Gravity**	**p=0.000****	**p=0.000****	**p=0.000****	**p=0.000****	**p=0.000****	\

* p<0.05, **p<0.01.
